# Poly[aqua­(μ_3_-2-hy­droxy-5-nitro­benzoato-κ^3^ *O* ^1^:*O* ^1′^:*O* ^2^)rubidium]

**DOI:** 10.1107/S1600536811037561

**Published:** 2011-09-17

**Authors:** Graham Smith, Urs D. Wermuth, Michael L. Williams

**Affiliations:** a Faculty of Science and Technology, Queensland University of Technology, GPO Box 2434, Brisbane, Queensland 4001, Australia; b School of Biomolecular and Physical Sciences, Griffith University, Nathan, Queensland 4111, Australia

## Abstract

In the structure of title compound, [Rb(C_7_H_4_NO_5_)(H_2_O)]_*n*_, the centrosymmetric cyclic dimeric repeating unit comprises two irregular RbO_4_ complex centres bridged by the carboxyl­ate groups of the 5-nitro­salicylate ligands. The coordination about each Rb atom is completed by a monodentate water mol­ecule and a phenolic O-atom donor which gives a bridging extension [Rb—O range = 3.116 (7)–3.135 (5) Å]. The polymeric structure is stabilized by inter­molecular water O—H⋯O_carboxyl­ate_ hydrogen bonds and weak inter-ring π–π inter­actions [minimum ring centroid separation = 3.620 (4) Å]. An intramolecular O—H⋯O hydrogen bond between phenol and carboxylate groups is also present.

## Related literature

For the structures of some Rb complexes with aromatic carb­oxy­lic acids, see: Dinnebier *et al.* (2002 ▶); Wiesbrock & Schmidbaur (2003 ▶); Smith *et al.* (2007 ▶). For the structure of 5-nitro­asalicylic acid and some Lewis base salts and metal complexes of this acid, see: Kumar *et al.* (2003 ▶); Smith *et al.* (2005 ▶); Morgant *et al.* (2006 ▶).
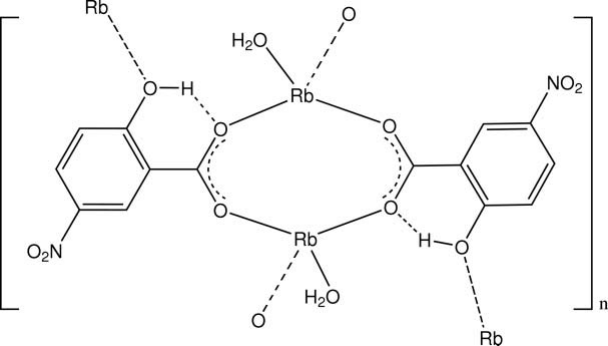


## Experimental

### 

#### Crystal data

[Rb(C_7_H_4_NO_5_)(H_2_O)]
*M* *_r_* = 285.6
Monoclinic, 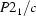

*a* = 11.9738 (5) Å
*b* = 12.0230 (4) Å
*c* = 6.9571 (3) Å
β = 105.401 (4)°
*V* = 965.59 (7) Å^3^
*Z* = 4
Mo *K*α radiation
μ = 5.14 mm^−1^
*T* = 200 K
0.50 × 0.20 × 0.10 mm

#### Data collection

Oxford Diffraction Gemini-S CCD-detector diffractometer
Absorption correction: multi-scan (*CrysAlis PRO*; Oxford Diffraction, 2010 ▶) *T*_min_ = 0.572, *T*_max_ = 0.980
6051 measured reflections
1893 independent reflections
1651 reflections with *I* > 2σ(*I*)
*R*_int_ = 0.027

#### Refinement

*R*[*F* ^2^ > 2σ(*F* ^2^)] = 0.054
*wR*(*F* ^2^) = 0.181
*S* = 1.17
1893 reflections
136 parameters
H-atom parameters constrained
Δρ_max_ = 0.81 e Å^−3^
Δρ_min_ = −1.11 e Å^−3^

### 

Data collection: *CrysAlis PRO* (Oxford Diffraction, 2010 ▶); cell refinement: *CrysAlis PRO*; data reduction: *CrysAlis PRO*; program(s) used to solve structure: *SIR92* (Altomare *et al.*, 1994 ▶); program(s) used to refine structure: *SHELXL97* (Sheldrick, 2008 ▶) within *WinGX* (Farrugia, 1999 ▶); molecular graphics: *PLATON* (Spek, 2009 ▶); software used to prepare material for publication: *PLATON*.

## Supplementary Material

Crystal structure: contains datablock(s) global, I. DOI: 10.1107/S1600536811037561/nk2111sup1.cif

Structure factors: contains datablock(s) I. DOI: 10.1107/S1600536811037561/nk2111Isup2.hkl

Additional supplementary materials:  crystallographic information; 3D view; checkCIF report

## Figures and Tables

**Table 1:** Hydrogen-bond geometry (Å, °)

*D*—H⋯*A*	*D*—H	H⋯*A*	*D*⋯*A*	*D*—H⋯*A*
O2—H2⋯O12	0.97	1.61	2.468 (8)	145
O1*W*—H11*W*⋯O11^i^	0.89	1.90	2.794 (9)	179
O1*W*—H12*W*⋯O12^ii^	0.90	1.96	2.861 (9)	180

Symmetry codes: (i) 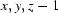; (ii) 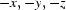.

**Fig. 1.**
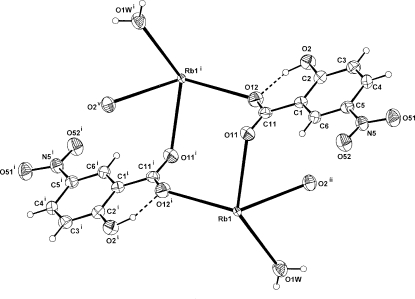
The molecular configuration and atom-numbering scheme for the dimeric repeat unit of the title complex, with non-H atoms drawn as 40% probability ellipsoids. For symmetry codes: (i) x, y, z - 1; (ii) -x, -y, -z; (v) -x, y - 1/2, -z + 3/2.

**Fig. 2.**
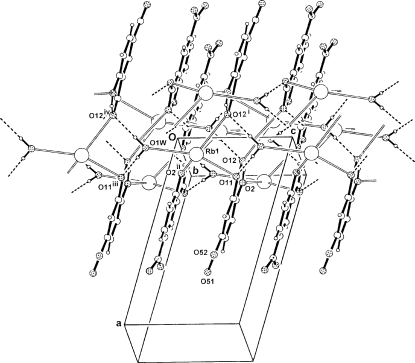
A perspective view of the polymeric structure with hydrogen bonds shown as dashed lines. For symmetry codes, see Fig. 1 and Table 1.

## supplementary crystallographic information

### Comment

The structures of the alkali metal complexes with aromatic carboxylic acids are of interest, particularly with the heavier metals Rb and Cs, because of their expanded coordination spheres and their ability to form polymeric systems. Although the structures of a series of metal^II^ complex adducts with 5-nitrosalicylic acid (5-NSA), of the type [*M*(5-NSA^-^)(H_2_O)_5_]. (5-NSA). H_2_O (*M* = Mg, Co, Ni, Zn) have been reported (Morgant *et al.*, 2006), no alkali metal complexes with 5-NSA are known. We obtained crystals of the title compound [Rb_2_(C_7_H_4_NO_2_)_2_(H_2_O)_2_]_n_ from the reaction of 5-NSA with rubidium hydroxide and the structure is reported here.

In the structure of this complex, the cyclic centrosymmetric dimeric repeating unit (Fig. 1) comprises two irregular RbO_4_ complex centres bridged by the carboxylate groups of the 5-NSA ligands. The coordination about each Rb is completed by a monodentate water molecule and a phenolic O donor which gives a bridging extension [Rb—O range 3.116 (7)–3.135 (5) Å]. The nitro O atoms (O51, O52) also give a weak symmetric bidentate association with inversion–related Rb centres [Rb—O, 3.290 (7), 3.261 (8) Å], a little too long to be considered formal Rb—O bonds. The coordination about Rb in this structure is therefore simpler than is found in other polymeric rubidium carboxylate complexes, *e.g.* and in rubidium salicylate (RbO_7_) (Dinnebier *et al.*, 2002) and rubidium anthranilate monohydrate (RbO_8_) (Wiesbrock & Schmidbaur, 2003) and rubidium sulfosalicylate 1.33 hydrate (RbO_7_) (Smith *et al.*, 2007).

The two-dimensional polymeric structure of the title compound (Fig. 2) is stabilized by intermolecular water *O*—*H*···O_carboxyl_ hydrogen bonds (Table 1) and weak inter-ring π–π interactions [minimum ring centroid separation, 3.620 (4) Å]. The 5-NSA anion has the short intramolecular phenolic *O*—*H*···O_carboxyl_ hydrogen bond and the essentially planar conformation commonly found in this ligand (Kumar *et al.*, 2003; Smith *et al.*, 2005) [torsion angles: C2—C1—C11—O11, -177.1 (7)°; C4—C5—N5—O52, 172.0 (7)°].

### Experimental

The title compound was synthesized by heating together under reflux for 15 minutes, 1 mmol quantities of 5-nitrosalicylic acid and rubidium hydroxide in 50 ml of 1:9 ethanol–water. After concentration to *ca* 30 ml, partial room temperature evaporation of the solution gave pale yellow needle prisms from which a suitable specimen was cleaved for the X-ray analysis.

### Refinement

The water and hydroxyl H atoms were located in a difference-Fourier synthesis and their positional and isotropic displacement parameters were allowed to ride together with the ring hydrogen atoms which were included in calculated positions with C—H = 0.93 Å and with *U*_iso_(H) = 1.2*U*_eq_(C,*O*).

### Crystal data

**Table:**

[Rb(C_7_H_4_NO_5_)(H_2_O)]	*F*(000) = 560
*M**_r_* = 285.60	*D*_x_ = 1.965 Mg m^−^^3^
Monoclinic, *P*2_1_/*c*	Mo *K*α radiation, λ = 0.71073 Å
Hall symbol: -P 2ybc	Cell parameters from 4363 reflections
*a* = 11.9738 (5) Å	θ = 3.4–28.8°
*b* = 12.0230 (4) Å	µ = 5.14 mm^−^^1^
*c* = 6.9571 (3) Å	*T* = 200 K
β = 105.401 (4)°	Needle, yellow
*V* = 965.59 (7) Å^3^	0.50 × 0.20 × 0.10 mm
*Z* = 4	

### Data collection

**Table:**

Oxford Diffraction Gemini-S CCD-detector diffractometer	1893 independent reflections
Radiation source: Enhance (Mo) X-ray source	1651 reflections with *I* > 2σ(*I*)
graphite	*R*_int_ = 0.027
Detector resolution: 16.077 pixels mm^-1^	θ_max_ = 26.0°, θ_min_ = 3.4°
ω scans	*h* = −14→14
Absorption correction: multi-scan (*CrysAlis PRO*; Oxford Diffraction, 2010)	*k* = −14→14
*T*_min_ = 0.572, *T*_max_ = 0.980	*l* = −8→8
6051 measured reflections	

### Refinement

**Table:**

Refinement on *F*^2^	Primary atom site location: structure-invariant direct methods
Least-squares matrix: full	Secondary atom site location: difference Fourier map
*R*[*F*^2^ > 2σ(*F*^2^)] = 0.054	Hydrogen site location: inferred from neighbouring sites
*wR*(*F*^2^) = 0.181	H-atom parameters constrained
*S* = 1.17	*w* = 1/[σ^2^(*F*_o_^2^) + (0.1295*P*)^2^ + 0.7347*P*] where *P* = (*F*_o_^2^ + 2*F*_c_^2^)/3
1893 reflections	(Δ/σ)_max_ < 0.001
136 parameters	Δρ_max_ = 0.81 e Å^−^^3^
0 restraints	Δρ_min_ = −1.11 e Å^−^^3^

### Special details

**Table:**

Geometry. Bond distances, angles *etc*. have been calculated using the rounded fractional coordinates. All su's are estimated from the variances of the (full) variance-covariance matrix. The cell e.s.d.'s are taken into account in the estimation of distances, angles and torsion angles
Refinement. Refinement of *F*^2^ against ALL reflections. The weighted *R*-factor *wR* and goodness of fit *S* are based on *F*^2^, conventional *R*-factors *R* are based on *F*, with *F* set to zero for negative *F*^2^. The threshold expression of *F*^2^ > σ(*F*^2^) is used only for calculating *R*-factors(gt) *etc*. and is not relevant to the choice of reflections for refinement. *R*-factors based on *F*^2^ are statistically about twice as large as those based on *F*, and *R*- factors based on ALL data will be even larger.

### Fractional atomic coordinates and isotropic or equivalent isotropic displacement parameters (Å^2^)

**Table:**

	*x*	*y*	*z*	*U*_iso_*/*U*_eq_	
Rb1	0.11224 (4)	−0.11709 (4)	0.23072 (7)	0.0208 (2)	
O1W	0.0713 (6)	−0.1033 (5)	−0.2300 (10)	0.068 (2)	
O2	0.1661 (5)	0.3786 (4)	0.5968 (10)	0.056 (2)	
O11	0.2015 (5)	0.0401 (5)	0.5983 (9)	0.063 (2)	
O12	0.0880 (4)	0.1881 (5)	0.5829 (8)	0.0583 (19)	
O51	0.6650 (5)	0.2709 (6)	0.5398 (9)	0.070 (2)	
O52	0.6074 (6)	0.1054 (5)	0.5877 (13)	0.068 (3)	
N5	0.5896 (5)	0.2061 (6)	0.5701 (9)	0.049 (2)	
C1	0.2840 (6)	0.2186 (6)	0.5897 (9)	0.041 (2)	
C2	0.2690 (6)	0.3339 (6)	0.5920 (10)	0.043 (2)	
C3	0.3609 (7)	0.4063 (7)	0.5906 (11)	0.049 (3)	
C4	0.4652 (7)	0.3660 (6)	0.5873 (12)	0.046 (2)	
C5	0.4801 (6)	0.2488 (6)	0.5818 (9)	0.043 (2)	
C6	0.3918 (6)	0.1769 (6)	0.5851 (10)	0.042 (2)	
C11	0.1863 (7)	0.1422 (6)	0.5922 (11)	0.043 (2)	
H2	0.10940	0.32000	0.58910	0.0670*	
H3	0.34960	0.48270	0.59180	0.0580*	
H4	0.52640	0.41390	0.58870	0.0560*	
H6	0.40380	0.10050	0.58420	0.0500*	
H11W	0.11310	−0.05780	−0.28540	0.0810*	
H12W	0.02110	−0.12980	−0.34140	0.0810*	

### Atomic displacement parameters (Å^2^)

**Table:**

	*U*^11^	*U*^22^	*U*^33^	*U*^12^	*U*^13^	*U*^23^
Rb1	0.0199 (4)	0.0188 (4)	0.0234 (4)	−0.0022 (2)	0.0050 (2)	0.0002 (2)
O1W	0.069 (4)	0.071 (4)	0.058 (4)	−0.022 (3)	0.009 (3)	0.002 (3)
O2	0.045 (3)	0.058 (4)	0.065 (4)	0.005 (2)	0.014 (3)	−0.003 (2)
O11	0.072 (4)	0.048 (4)	0.074 (4)	−0.007 (3)	0.027 (3)	−0.008 (3)
O12	0.045 (3)	0.071 (4)	0.059 (3)	−0.002 (3)	0.014 (2)	−0.005 (3)
O51	0.053 (3)	0.071 (4)	0.090 (4)	−0.007 (3)	0.026 (3)	0.003 (4)
O52	0.057 (4)	0.050 (4)	0.099 (5)	0.012 (3)	0.024 (4)	0.007 (3)
N5	0.043 (3)	0.047 (4)	0.056 (4)	−0.002 (3)	0.012 (3)	0.004 (3)
C1	0.045 (4)	0.042 (4)	0.035 (3)	0.000 (3)	0.010 (3)	−0.005 (3)
C2	0.043 (4)	0.046 (4)	0.040 (4)	0.005 (3)	0.013 (3)	0.003 (3)
C3	0.058 (5)	0.042 (4)	0.047 (4)	0.005 (3)	0.015 (4)	0.002 (3)
C4	0.050 (4)	0.044 (4)	0.047 (4)	−0.009 (3)	0.016 (3)	0.002 (3)
C5	0.044 (4)	0.044 (4)	0.039 (3)	0.004 (3)	0.009 (3)	0.000 (3)
C6	0.048 (4)	0.039 (4)	0.038 (3)	0.000 (3)	0.011 (3)	0.002 (3)
C11	0.046 (4)	0.044 (4)	0.039 (4)	−0.003 (3)	0.010 (3)	−0.009 (3)

### Geometric parameters (Å, °)

**Table:**

Rb1—O1W	3.116 (7)	N5—C5	1.430 (10)
Rb1—O11	3.131 (6)	C1—C11	1.491 (11)
Rb1—O12^i^	3.132 (5)	C1—C2	1.399 (10)
Rb1—O2^ii^	3.135 (5)	C1—C6	1.393 (10)
O2—C2	1.353 (10)	C2—C3	1.405 (11)
O11—C11	1.240 (9)	C3—C4	1.345 (12)
O12—C11	1.286 (10)	C4—C5	1.422 (10)
O51—N5	1.252 (9)	C5—C6	1.371 (10)
O52—N5	1.230 (9)	C3—H3	0.9300
O1W—H11W	0.8900	C4—H4	0.9300
O1W—H12W	0.9000	C6—H6	0.9300
O2—H2	0.9700		
			
O1W—Rb1—O11	137.27 (16)	C6—C1—C11	120.9 (7)
O1W—Rb1—O12^i^	120.79 (17)	C1—C2—C3	120.7 (7)
O1W—Rb1—O2^ii^	68.54 (16)	O2—C2—C3	118.3 (7)
O11—Rb1—O12^i^	87.66 (15)	O2—C2—C1	121.0 (6)
O2^ii^—Rb1—O11	68.78 (16)	C2—C3—C4	120.6 (7)
O2^ii^—Rb1—O12^i^	127.80 (16)	C3—C4—C5	118.8 (7)
Rb1—O2^ii^—C2^ii^	129.2 (4)	N5—C5—C6	119.8 (7)
Rb1—O11—C11	123.7 (5)	C4—C5—C6	121.4 (7)
Rb1^i^—O12—C11	130.8 (5)	N5—C5—C4	118.7 (7)
Rb1—O1W—H11W	121.00	C1—C6—C5	119.8 (7)
Rb1—O1W—H12W	139.00	O11—C11—C1	120.1 (7)
H11W—O1W—H12W	100.00	O12—C11—C1	116.5 (6)
Rb1^iii^—O2—H2	119.00	O11—C11—O12	123.4 (8)
C2—O2—H2	110.00	C2—C3—H3	120.00
O51—N5—C5	119.9 (7)	C4—C3—H3	120.00
O52—N5—C5	119.0 (7)	C3—C4—H4	121.00
O51—N5—O52	121.1 (7)	C5—C4—H4	121.00
C2—C1—C6	118.7 (7)	C1—C6—H6	120.00
C2—C1—C11	120.5 (7)	C5—C6—H6	120.00
			
O1W—Rb1—O11—C11	32.4 (7)	C2—C1—C6—C5	−0.4 (9)
O12^i^—Rb1—O11—C11	−102.9 (6)	C11—C1—C6—C5	179.7 (6)
O2^ii^—Rb1—O11—C11	29.5 (6)	C2—C1—C11—O11	−177.1 (7)
O1W—Rb1—O12^i^—C11^i^	−62.5 (6)	C2—C1—C11—O12	4.5 (10)
O11—Rb1—O12^i^—C11^i^	83.8 (6)	C6—C1—C11—O11	2.9 (10)
O1W—Rb1—O2^ii^—C2^ii^	−96.4 (6)	C6—C1—C11—O12	−175.5 (6)
O11—Rb1—O2^ii^—C2^ii^	81.5 (6)	C11—C1—C2—O2	0.1 (10)
Rb1—O2^ii^—C2^ii^—C1^ii^	−159.4 (5)	C11—C1—C2—C3	179.7 (6)
Rb1—O2^ii^—C2^ii^—C3^ii^	20.2 (9)	C6—C1—C2—O2	−179.9 (6)
Rb1—O11—C11—O12	73.6 (9)	C6—C1—C2—C3	−0.3 (10)
Rb1—O11—C11—C1	−104.7 (7)	O2—C2—C3—C4	179.5 (7)
Rb1^i^—O12—C11—O11	34.3 (11)	C1—C2—C3—C4	−0.1 (11)
Rb1^i^—O12—C11—C1	−147.4 (5)	C2—C3—C4—C5	1.1 (11)
O51—N5—C5—C4	−8.9 (9)	C3—C4—C5—N5	177.5 (7)
O52—N5—C5—C4	172.0 (7)	C3—C4—C5—C6	−1.7 (11)
O51—N5—C5—C6	170.3 (6)	N5—C5—C6—C1	−177.8 (6)
O52—N5—C5—C6	−8.8 (10)	C4—C5—C6—C1	1.4 (10)

Symmetry codes: (i) −*x*, −*y*, −*z*+1; (ii) *x*, −*y*+1/2, *z*−1/2; (iii) *x*, −*y*+1/2, *z*+1/2.

### Hydrogen-bond geometry (Å, °)

**Table:**

*D*—H···*A*	*D*—H	H···*A*	*D*···*A*	*D*—H···*A*
O2—H2···O12	0.97	1.61	2.468 (8)	145
O1W—H11W···O11^iv^	0.89	1.90	2.794 (9)	179
O1W—H12W···O12^v^	0.90	1.96	2.861 (9)	180

Symmetry codes: (iv) *x*, *y*, *z*−1; (v) −*x*, −*y*, −*z*.
